# Supplementary material for: New particle formation in the remote marine boundary layer
Source: Nat Commun. 2021 Jan 22;12:527. doi: 10.1038/s41467-020-20773-1 (PMC7822916; doi:10.1038/s41467-020-20773-1)
Supplement: Supplementary file 1 — Supplementary Information [file 41467_2020_20773_MOESM1_ESM.pdf]

Supplementary Information for:

New particle formation in the remote marine boundary layer

Authors

Guangjie Zheng[1,2†], Yang Wang[1,3†], Robert Wood[4], Michael P. Jensen[2], Chongai Kuang[2], Isabel L. McCoy[4], Alyssa Matthews[5], Fan Mei[5], Jason M. Tomlinson[5], John E. Shilling[5], Maria A. Zawadowicz[5], Ewan Crosbie[6,7], Richard Moore[6], Luke Ziemba[6], Meinrat O. Andreae[8,9], and Jian Wang[1,2*]

Affiliations

[1]Center for Aerosol Science and Engineering, Department of Energy, Environmental and Chemical Engineering, Washington University in St. Louis, St. Louis, Missouri, USA

[2]Environmental and Climate Science Department, Brookhaven National Laboratory, Upton, New York, USA

[3]Department of Civil, Architectural and Environmental Engineering, Missouri University of Science and Technology, Rolla, Missouri, USA

[4]Department of Atmospheric Science, University of Washington, Seattle, Washington, USA

[5]Atmospheric Measurement & Data Sciences, Pacific Northwest National Laboratory, Richland, Washington, USA

[6]NASA Langley Research Center, Hampton, Virginia, USA

[7]Science Systems and Applications, Inc., Hampton, Virginia, USA

[8]Max Planck Institute for Chemistry, Mainz, Germany

[9]Scripps Institution of Oceanography, University of California San Diego, La Jolla, California, USA

†These authors contribute equally to this work.

***Correspond to:** J.W. (jian@wustl.edu)

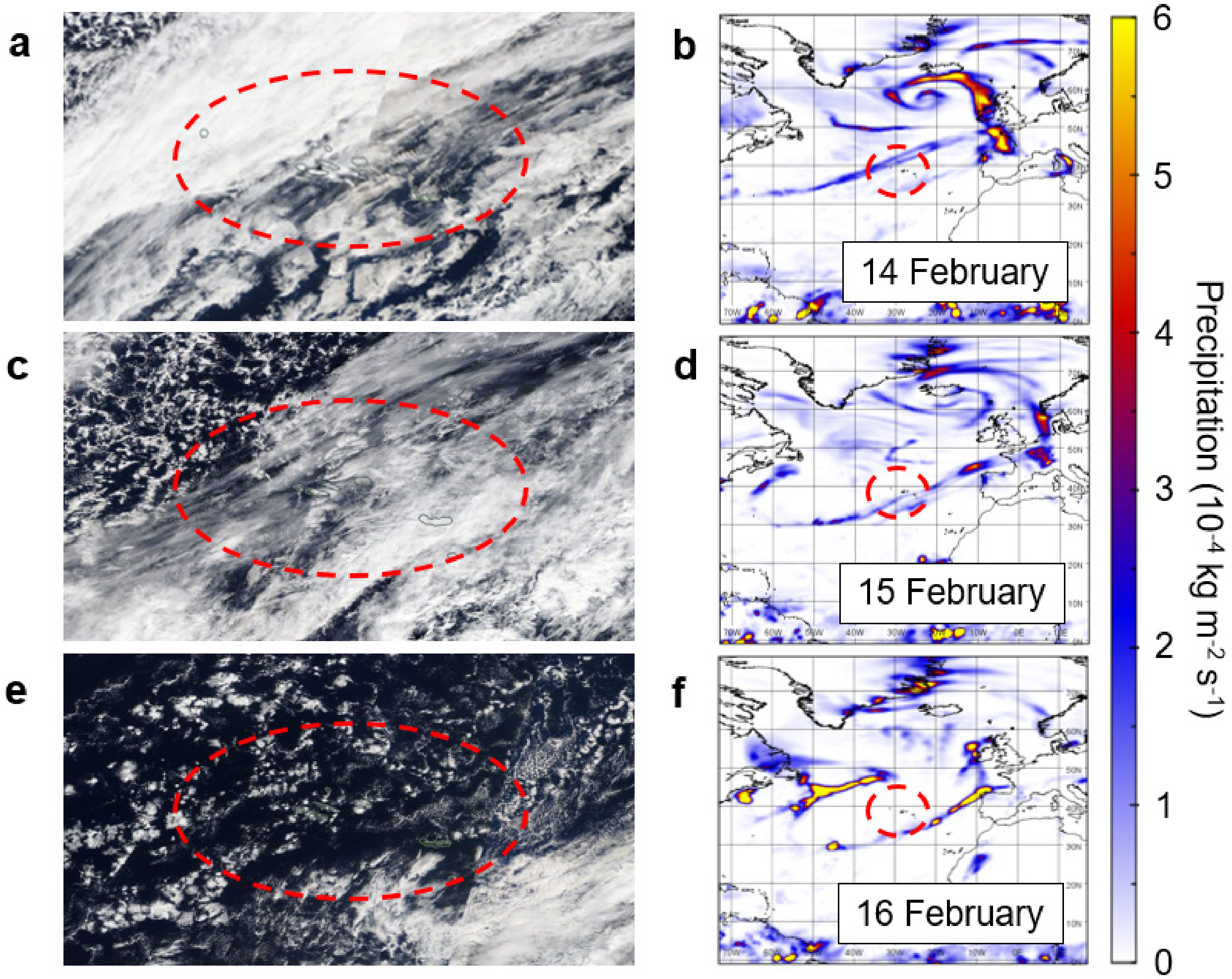

Supplementary Figure 1. Cold air outbreak event from 14 to 16 February 2018. (a), **(c)**, and **(e)** MODIS satellite images (obtained from NASA Worldview) showing the movement of a cold front and the open cellular convection behind it. **(b)**, **(d)**, and **(f)** Precipitation data from Modern-Era Retrospective Analysis for Research and Applications, Version 2 (MERRA-2)[1], in 10^{-4} kg m^{-2} s^{-1}. The location of the Azores Islands is highlighted by red dashed circles.

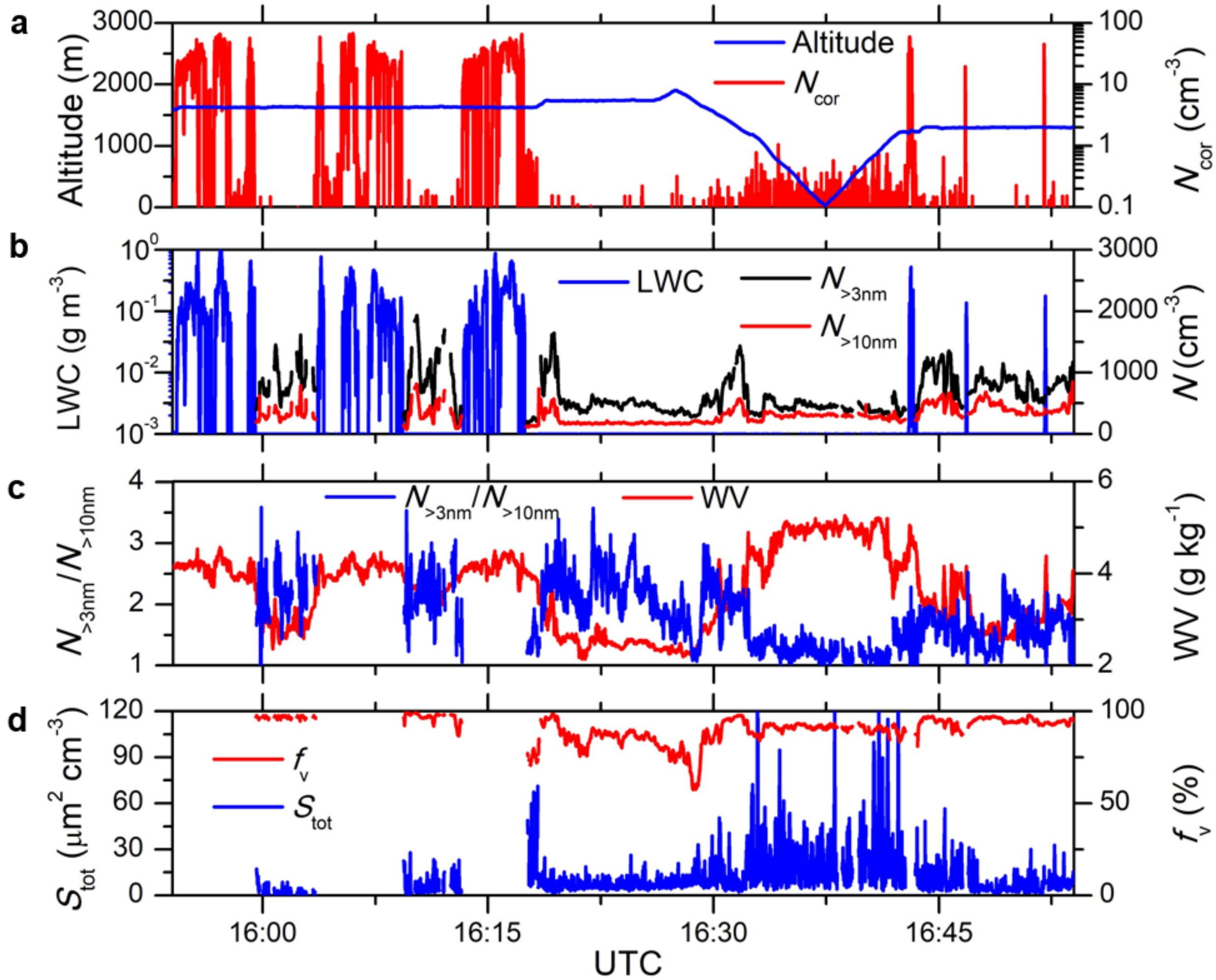

Supplementary Figure 2. Time series of relevant measurements onboard the Gulfstream-1 aircraft during the marine boundary layer new particle formation event observed on 16 February 2018. (a) G-1 altitude and the number concentration of coarse mode aerosol (i.e., the concentration of particles with diameter larger than 1 µm, N_{cor}). (**b**) Liquid water content (LWC) and number concentration of particles larger than 3 nm ($N_{>3nm}$) and 10 nm ($N_{>10nm}$). The number concentrations are normalized to standard temperature and pressure (273.15 K and 101.325 kPa). **(c)** $N_{>3nm}/N_{>10nm}$ and water vapor mixing ratio (WV). **(d)** Total ambient particle surface area concentration (S_{tot}) and the number fraction of particles volatile at 300 ºC (f_v). Source data are provided as a Source Data file.

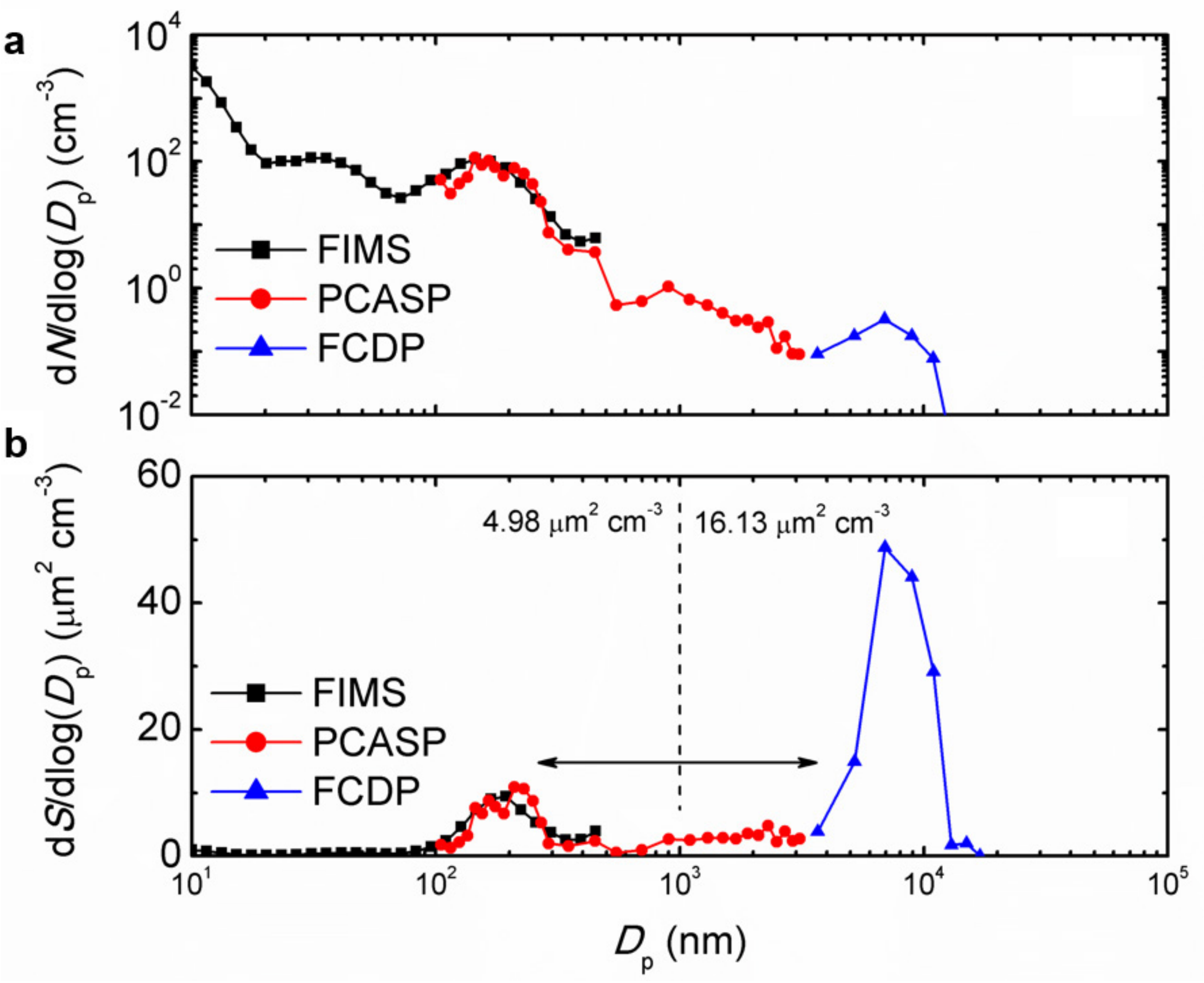

Supplementary Figure 3. Particle size distributions measured in the surface mixed layer during the G-1 flight on 16 February 2018 between 16:33 and 16:35 UTC. (a) Number-based size distributions. **(b)** Surface area-based size distributions. The integrated surface area concentrations for submicron and supermicron particles are 4.98 and 16.13 μm^2 cm^{-3}, respectively. The particle size distributions were measured with a fast-integrated mobility spectrometer (FIMS, size range of 10 to 500 nm), a passive cavity aerosol spectrometer probe (PCASP, size range of 0.1 to 3 μm), and a fast cloud droplet probe (FCDP, size range of 2.5 to 50 μm). The dominance of supermicron particles in the surface area concentration indicates a strong contribution from sea spray aerosol. Source data are provided as a Source Data file.

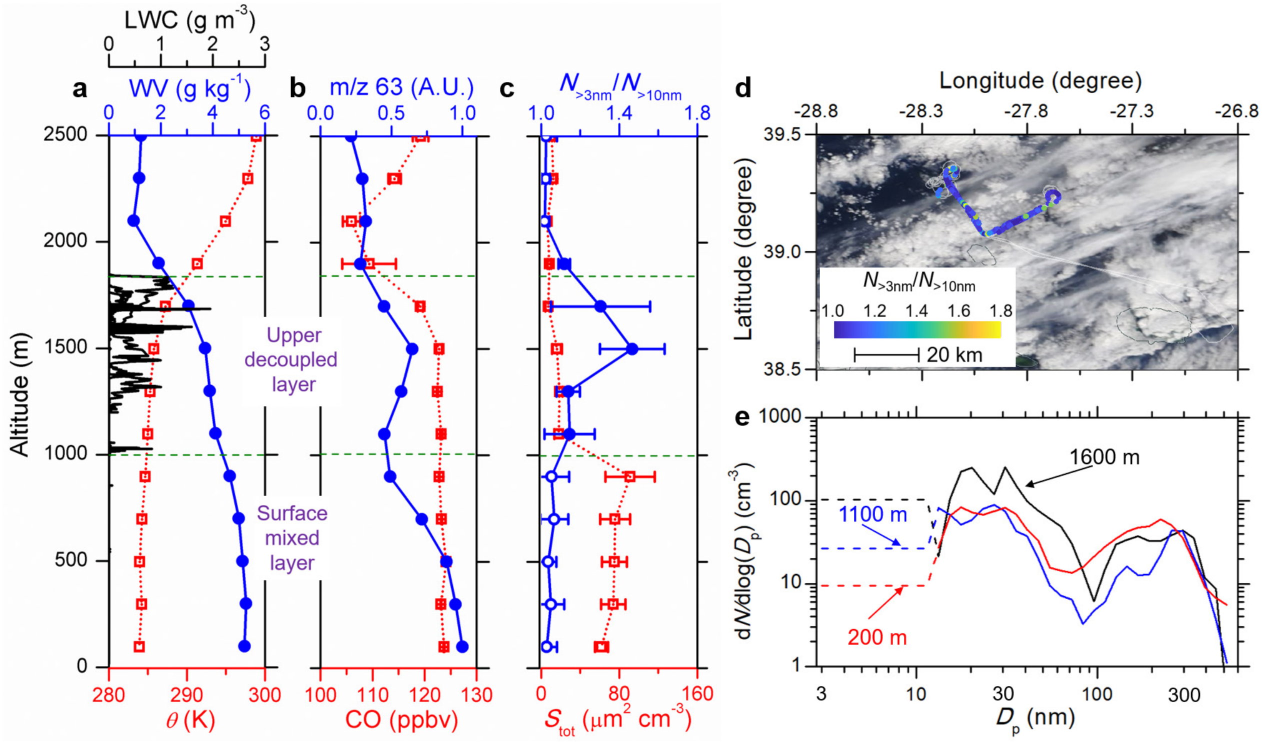

Supplementary Figure 4. Same as Fig. 1 for measurements onboard the G-1 aircraft upwind of the Eastern North Atlantic (ENA) site on 24 January 2018. (a) Vertical profiles of potential temperature (θ), water vapor mixing ratio (WV, g H_2O / kg wet air), and liquid water content (LWC). **(b)** Vertical profiles of carbon monoxide (CO) and the ion signal (arbitrary units, A.U.) at m/z 63 (e.g., DMS). The error bars for CO represent one standard deviations of 1-s measurements. **(c)** Vertical profiles of the concentration ratio of particles larger than 3 nm to particles larger than 10 nm ($N_{>3nm}/N_{>10nm}$) and total particle surface area concentration (S_{tot}). Elevated $N_{>3nm}/N_{>10nm}$ (i.e., greater than 1.1) that are statistically significant for the altitude bins (detailed in Methods section) are marked with filled circles and the rest are shown by open circles. The error bars represent one standard deviations for 1 s $N_{>3nm}/N_{>10nm}$ and 10 s S_{tot} values, respectively. **(d)** Flight track of the G-1 aircraft during horizontal legs in the upper decoupled layer colored by $N_{>3nm}/N_{>10nm}$. During the flight, the wind was from the northwest. The flight tracks include both 30 km along-wind legs upwind of the ENA site and 30 km crosswind legs towards the northeast over the ocean, therefore the potential influence of island sources on G-1 aerosol measurements is negligible. The background image from NASA Worldview, taken by MODIS at an earlier time than the flight on the same day, is used to illustrate the cloud field. **(e)** Particle size distributions measured at three different altitudes within the marine boundary layer. The particle concentrations below 10 nm (shown by dashed lines) are derived as the difference between $N_{>3nm}$ measured by the CPC and the concentration of particles larger than 10 nm integrated from FIMS size distribution. The vertical profiles shown in (a) and (b) are based on measurements from 16:16 to 16:23 UTC. The liquid

water content during the entire flight is shown to illustrate the vertical extent of clouds. Source data are provided as a Source Data file.

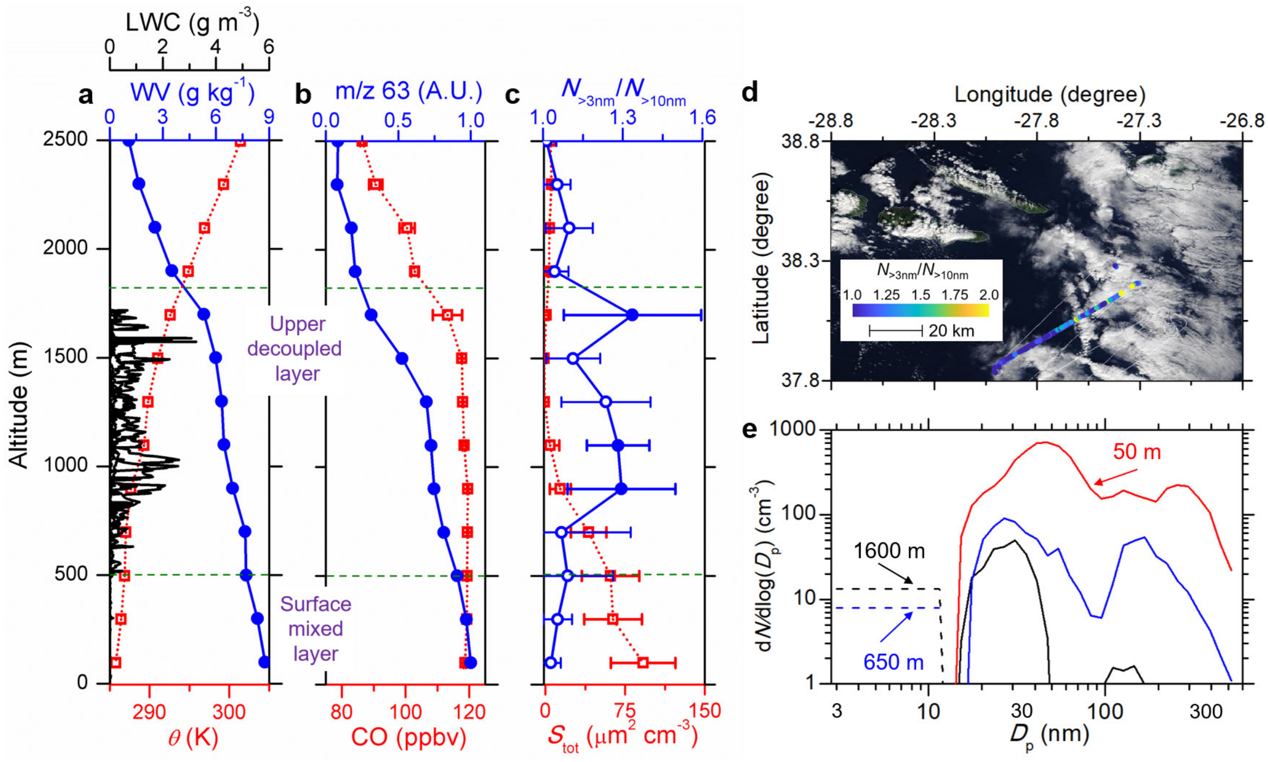

Supplementary Figure 5. Same as Fig. 1 for measurements onboard the G-1 aircraft on 28 January 2018. (a) Vertical profiles of potential temperature (θ), water vapor mixing ratio (WV, g H_2O / kg wet air), and liquid water content (LWC). **(b)** Vertical profiles of carbon monoxide (CO) and the ion signal (arbitrary units, A.U.) at m/z 63 (e.g., DMS). The error bars for CO represent one standard deviations of 1-s measurements. **(c)** Vertical profiles of the concentration ratio of particles larger than 3 nm to particles larger than 10 nm ($N_{>3nm}/N_{>10nm}$) and total particle surface area concentration (S_{tot}). Elevated $N_{>3nm}/N_{>10nm}$ (i.e., greater than 1.1) that are statistically significant for the altitude bins (detailed in Methods section) are marked with filled circles and the rest are shown by open circles. The error bars represent one standard deviations for 1 s $N_{>3nm}/N_{>10nm}$ and 10 s S_{tot} values, respectively. **(d)** Flight track of the G-1 aircraft during horizontal legs in the upper decoupled layer colored by $N_{>3nm}/N_{>10nm}$. During the flight, the wind was from the southeast (i.e., 140 degrees). The flight track was south of Terceira Island and potential influence of island sources on G-1 aerosol measurements is negligible. The background image from NASA Worldview, taken by MODIS at a time during the flight, is used to illustrate the scattered cloud field. **(e)** Particle size distributions measured at three different altitudes within the marine boundary layer. The particle concentrations below 10 nm (shown by dashed lines) are derived as the difference between $N_{>3nm}$ measured by the CPC and the concentration of particles larger than 10 nm integrated from the FIMS size distribution. The vertical profiles shown in (a) and (b) are based on measurements from 9:59 to 10:07 UTC. The liquid water content during the entire flight is shown to illustrate the vertical extent of clouds. Source data are provided as a Source Data file.

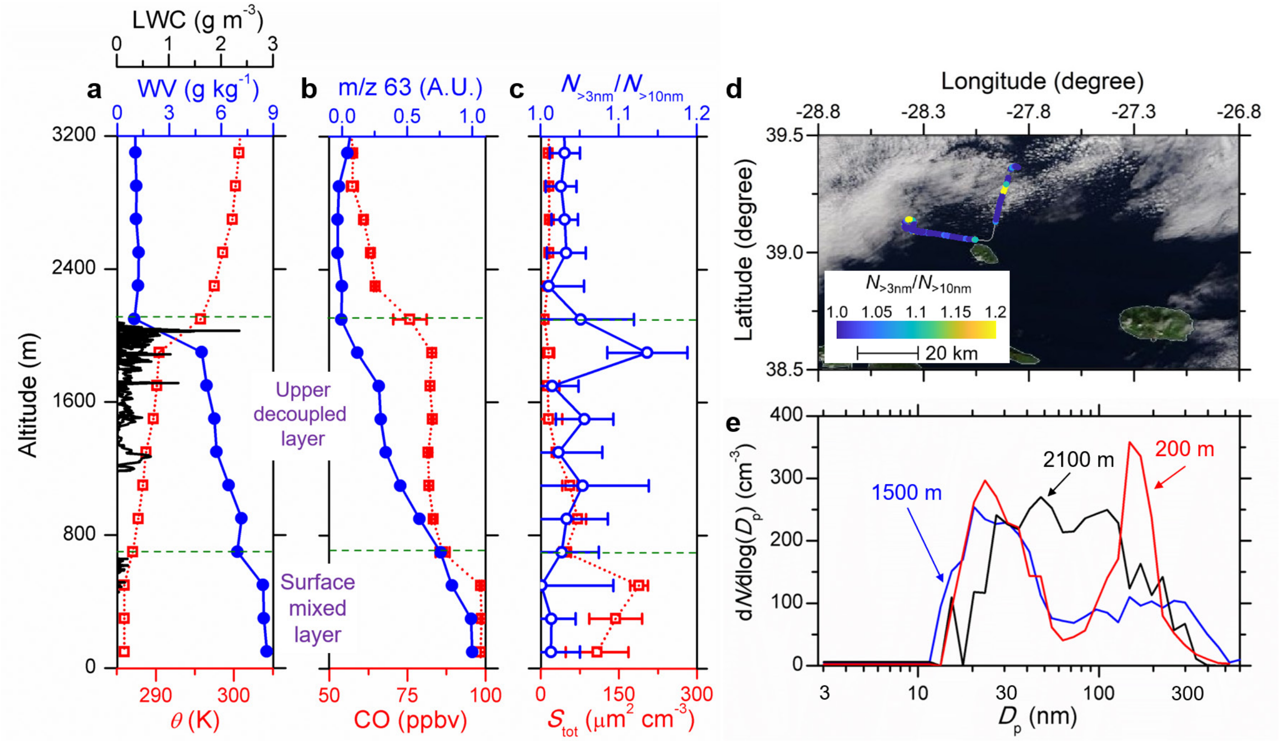

Supplementary Figure 6. Same as Fig. 1 for measurements onboard the G-1 aircraft upwind of the Eastern North Atlantic (ENA) site on 8 February 2018. (a) Vertical profiles of potential temperature (θ), water vapor mixing ratio (WV, g H_2O / kg wet air), and liquid water content (LWC). **(b)** Vertical profiles of carbon monoxide (CO) and the ion signal (arbitrary units, A.U.) at m/z 63 (e.g., DMS). The error bars for CO represent one standard deviations of 1-s measurements. **(c)** Vertical profiles of the concentration ratio of particles larger than 3 nm to particles larger than 10 nm ($N_{>3nm}/N_{>10nm}$) and total particle surface area concentration (S_{tot}). Elevated $N_{>3nm}/N_{>10nm}$ (i.e., greater than 1.1) that are statistically significant for the altitude bins (detailed in Methods section) are marked with filled circles and the rest are shown by open circles. The error bars represent one standard deviations for 1 s $N_{>3nm}/N_{>10nm}$ and 10 s S_{tot} values, respectively. **(d)** Flight track of the G-1 aircraft during horizontal legs in the upper decoupled layer colored by $N_{>3nm}/N_{>10nm}$. During the flight, the wind was from the west. The flight tracks include both 30 km along-wind legs upwind of the ENA site and 30 km crosswind legs towards the north over the ocean, therefore the potential influence of island sources on G-1 aerosol measurements is negligible. The background image from NASA Worldview, taken by MODIS on the same day, is used to illustrate the scattered cloud field. **(e)** Particle size distributions measured at three different altitudes, two inside marine boundary layer (i.e., 200 m and 1500 m), and one just above the inversion (i.e., 2100 m). The vertical profiles shown in (a) and (b) are based on measurements from 13:05 to 13:22 UTC. The liquid water content during the entire flight is shown to illustrate the vertical extent of clouds. Note that the nucleation mode particles in the upper decoupled layer (1500 m) exhibited a substantially smaller mode diameter than the aerosol immediately above the MBL (2100 m), suggesting recent

new particle formation in the upper part of marine boundary layer. Source data are provided as a Source Data file.

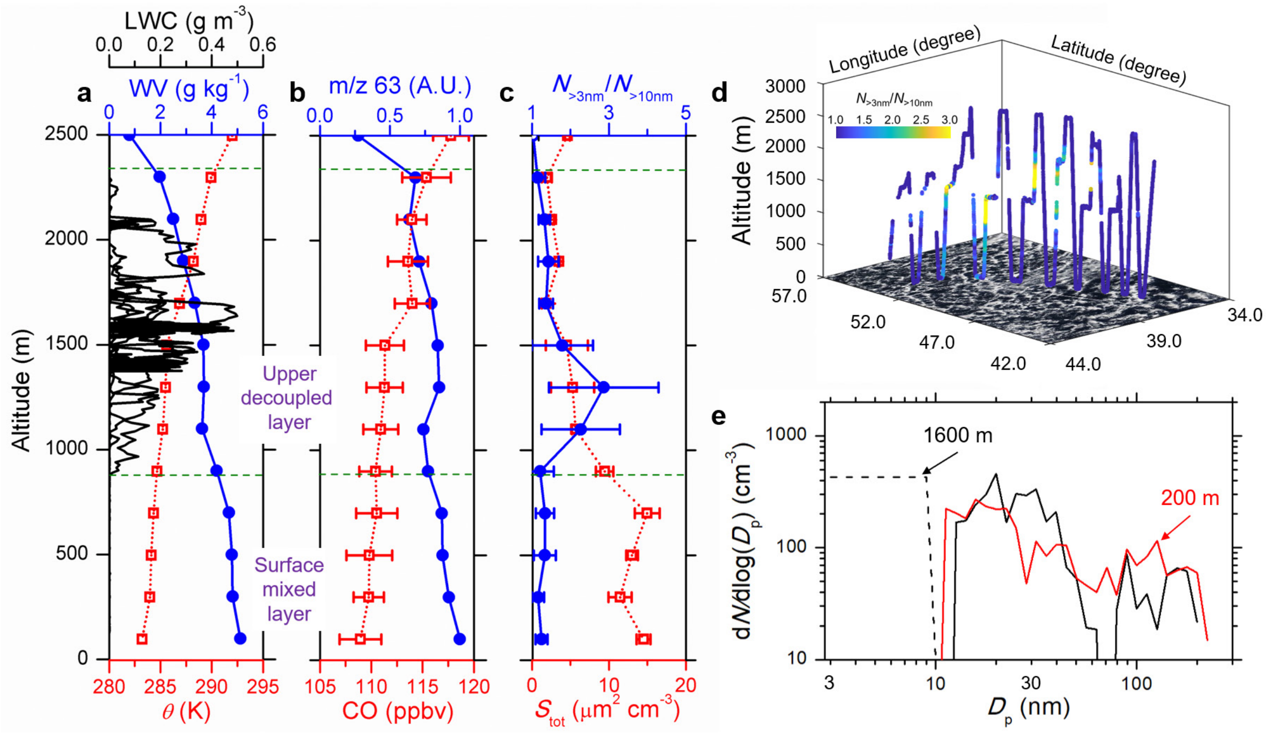

Supplementary Figure 7. Measurements onboard NASA C-130 aircraft on 19 September 2017 during the North Atlantic Aerosols and Marine Ecosystems Study (NAAMES). (a) Vertical profiles of potential temperature (θ), water vapor mixing ratio (WV, g H_2O / kg wet air), and liquid water content (LWC). **(b)** Vertical profiles of carbon monoxide (CO) and dimethyl sulfide (DMS) mixing ratios. The error bars for CO represent one standard deviations of 1-s measurements. **(c)** Vertical profiles of the concentration ratio of particles larger than 3 nm to particles larger than 10 nm ($N_{>3nm}/N_{>10nm}$) and total particle surface area concentration (S_{tot}). Elevated $N_{>3nm}/N_{>10nm}$ (i.e., greater than 1.1) that are statistically significant for the altitude bins (detailed in Methods section) are marked with filled circles and the rest are shown by open circles. The error bars represent one standard deviations for 1 s $N_{>3nm}/N_{>10nm}$ and 10 s S_{tot} values, respectively. **(d)** Flight track of C-130 over the North Atlantic Ocean colored by $N_{>3nm}/N_{>10nm}$. The background image from NASA Worldview, taken by MODIS on the same day, is used to illustrate the open-cell cloud field. **(e)** Particle size distributions measured at two different altitudes within the marine boundary layer by a scanning mobility particle sizer. The particle concentrations below 10 nm (shown by dashed lines) are derived as the difference between $N_{>3nm}$ measured by the CPC and the concentration of particles larger than 10 nm integrated from the SMPS size distribution. The vertical profiles shown in (a) and (b) are based on measurements from 16:26 to 16:42 UTC. The liquid water content during the entire flight is shown to illustrate the vertical extent of clouds. Source data are provided as a Source Data file.

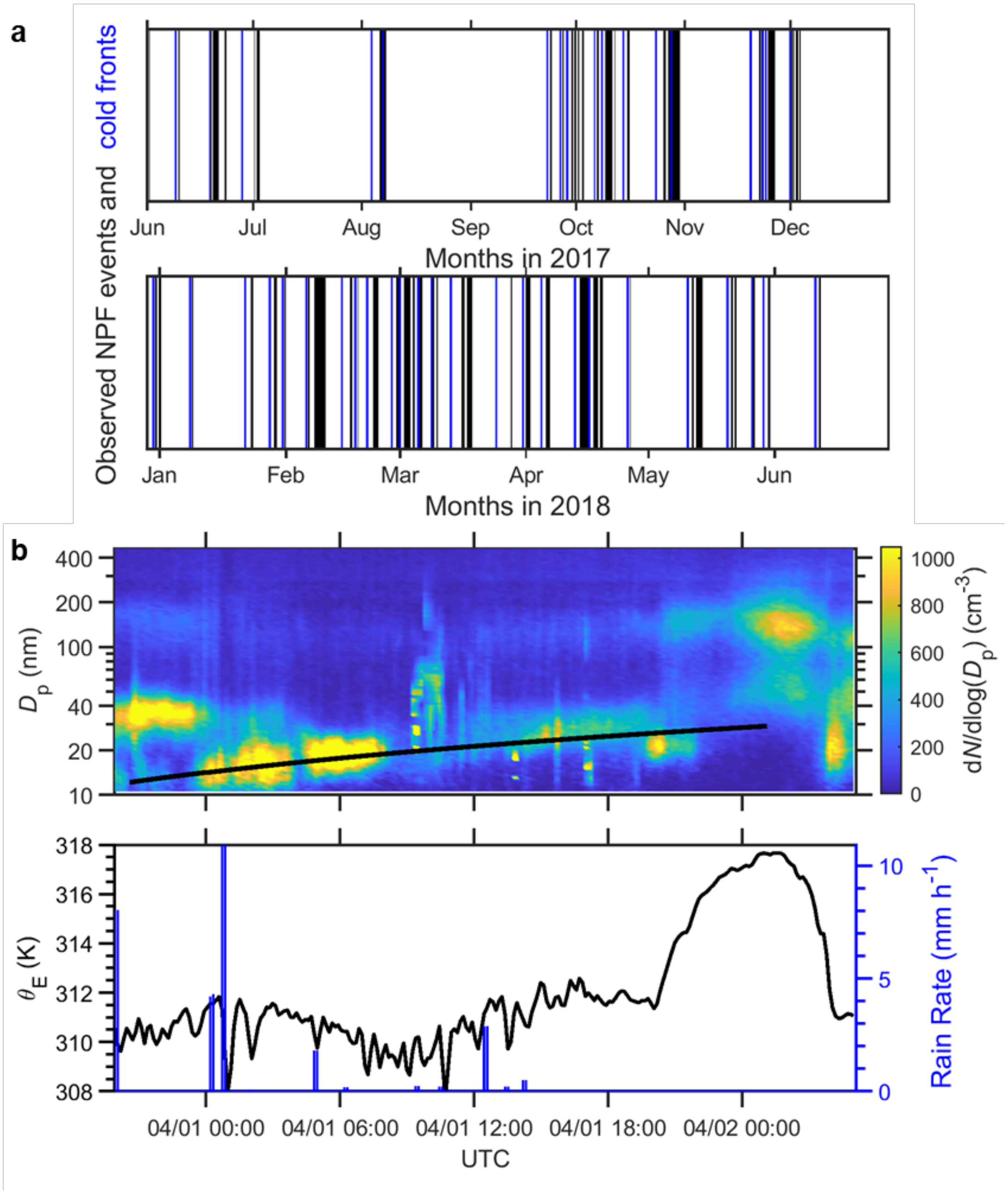

Supplementary Figure 8. Observed nucleation mode particle growth events from June 2017 to June 2018. (A) Event occurrences and the time for corresponding cold front passages prior to the events. The black lines represent the time of events when the growth of nucleation mode particles was observed at the ENA site. The blue lines indicate the time of corresponding cold front passages prior to the growth events. **(B)** An example of the growth events shown in (A). The aerosol size distribution measured by the SMPS (upper panel), equivalent potential temperature (θ_E) and rain rate (lower panel) are shown. A cold front passed the ENA site around 06:00 UTC on 31 March 2018. Following strong precipitation, nucleation mode particles with initial mode

diameter of ~ 10 nm emerged around 20:30 UTC on that day and exhibited growth over the next ~28 hours.

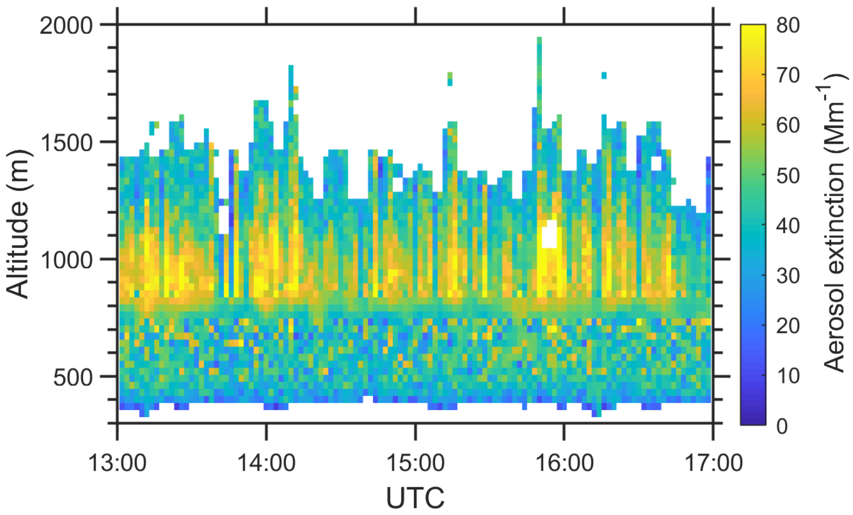

Supplementary Figure 9. Vertical profile of aerosol extinction from 13:00 to 17:00 UTC on 16 February 2018, retrieved from Raman lidar measurements over the ENA site. The aerosol extinction decreased with height from the top of surface mixed layer (~ 1050 m) into the upper decoupled layer, in agreement with the clean layer with reduced surface area concentration at ~1500 m observed onboard the G-1 aircraft during the MBL NPF event on the same day.

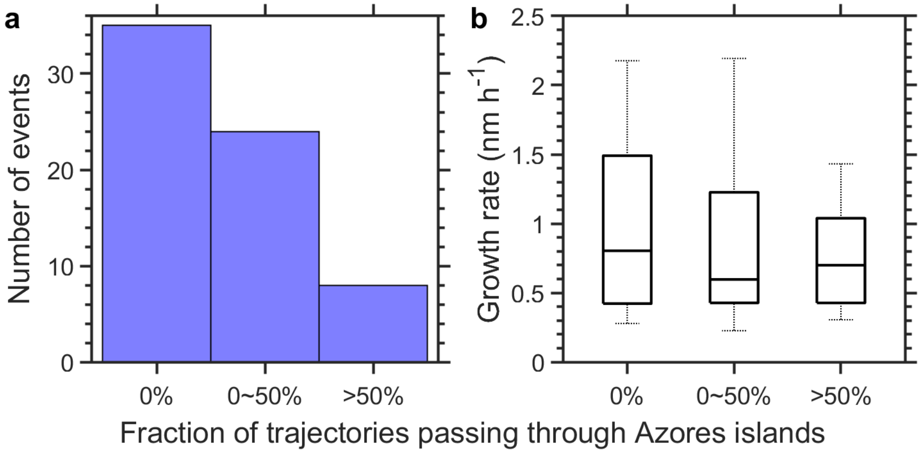

Supplementary Figure 10. Particle growth rate is independent of the fraction of the air masses passing through the Azores islands during the growth events. (a) Numbers of events with 0%, 0-50%, and >50% of the hourly trajectories passing through at least one of the Azores islands, and (b) statistics of particle growth rate for the different fractions. The box-whisker plot is drawn for 10-, 25-, 50-, 75-, and 90-percentiles.

Supplementary References

1 Gelaro, R. *et al.* The Modern-Era Retrospective Analysis for Research and Applications, Version 2 (MERRA-2). *Journal of Climate* **30**, 5419-5454, doi:10.1175/jcli-d-16-0758.1 (2017).
