# Supplementary material for: New particle formation in the remote marine boundary layer
Source: Nat Commun. 2021 Jan 22;12:527. doi: 10.1038/s41467-020-20773-1 (PMC7822916; doi:10.1038/s41467-020-20773-1)
Supplement: Supplementary file 3 — Description of Additional Supplementary Files [file 41467_2020_20773_MOESM3_ESM.pdf]

**Description of Additional Supplementary Files**

File Name: Supplementary Data 1
Description: **List of nucleation mode particle growth events observed at the ENA site from June 2017 to June 2018.** Events with G-1 flights on the same day are shaded.
